# Cost-effectiveness analysis of malaria rapid diagnostic test in the elimination setting

**DOI:** 10.1186/s40249-020-00745-9

**Published:** 2020-09-29

**Authors:** Yan-Qiu Du, Xiao-Xiao Ling, Jia-Jie Jin, Hua-Yun Zhou, Si Zhu, Guo-Ding Zhu, Wei Wang, Jun Cao, Jia-Yan Huang

**Affiliations:** 1 Key Lab of Health Technology Assessment, National Health Commission, School of Public Health, Fudan University, Shanghai, 200433 China; 2 Department of Statistical Science, University College London, WC1E 6BT, London, UK; 3 National Health Commission Key Laboratory of Parasitic Disease Control and Prevention, Jiangsu Provincial Key Laboratory of Parasite and Vector Control Technology, Jiangsu Institute of Parasitic Diseases, Wuxi, 214064 China; 4 Center for Global Health, School of Public Health, Nanjing Medical University, Nanjing, 211166 China; 5 Public Health Research Center, Jiangnan University, Wuxi, 214122 China

**Keywords:** Cost-effectiveness analysis, Monte Carlo simulation, Malaria elimination, Rapid diagnostic test, Microscopy

## Abstract

**Background:**

As more and more countries approaching the goal of malaria elimination, malaria rapid diagnostic tests (RDT) was recomendated to be a diagnostic strategy to achieve and maintain the statute of malaria free, as it’s less requirments on equipment and experitise than microscopic examination. But there are very few economic evaluations to confirm whether RDT was cost-effective in the setting of malaria elimination. This research aimed to offer evidence for helping decision making on malaria diagnosis strategy.

**Methods:**

A cost-effectiveness analysis was conducted to compare RDT with microscopy examination for malaria diagnosis, by using a decision tree model. There were three strategies of malaria diagnostic testing evaluated in the model, 1) microscopy, 2) RDT, 3) RDT followed by microscopy. The effect indicator was defined as the number of malaria cases treated appropriately. Based on the joint perspective of health sector and patient, costs data were collected from hospital information systems, key informant interviews, and patient surveys. Data collection was conducted in Jiangsu from September 2018 to January 2019. Epidemiological data were obtained from local malaria surveillance reports. A hypothetical cohort of 300 000 febrile patients were simulated to calculate the total cost and effect of each strategy. One-way, two-way, and probabilistic sensitivity analysis were performed to test the robustness of the result.

**Results:**

The results showed that RDT strategy was the most effective (245 cases) but also the most costly (United States Dollar [USD] 4.47 million) compared to using microscopy alone (238 cases, USD 3.63 million), and RDT followed by microscopy (221 cases, USD 2.75 million). There was no strategy dominated. One-way sensitivity analysis reflected that the result was sensitive to the change in labor cost and two-way sensitivity analysis indicated that the result was not sensitive to the proportion of *falciparum* malaria*.* The result of Monte Carlo simulation showed that RDT strategy had higher effects and higher cost than other strategies with a high probability.

**Conclusions:**

Compared to microscopy and RDT followed by microscopy, RDT strategy had higher effects and higher cost in the setting of malaria elimination.

## Background

Malaria is a parasitic disease caused by Plasmodium spp.*,* which is transmitted to human through the bites of infected anopheline mosquitoes. Although the malaria incidence rate declined globally from 71 to 57 cases per 1000 population at risk between 2010 and 2018 [1]. The global decrease trend appeared to slow from 2014 to 2018 [2]. China continued to make progress on elimination, and reported zero indigenous case since 2017. But here were still about 2500 imported cases [1, 3], and falciparum malaria accounted for more than 85% of them [4].

Malaria diagnostic tests (MDT), such as microscopy (microscopic examination of Giemsa-stained thick and thin blood films) and rapid diagnostic tests (RDT), are now recommended as routine diagnostic methods by the World Health Organization (WHO) in all suspected malaria patients before treatment [5]. Microscopy, as the conventional laboratory method for malaria diagnosis, needs to be conducted by microscopists with adequate training and essential equipment will also be required. It allows the differentiations of species and stages and the quantification of parasites. However, microscopy examination can have a high proportion of false negatives due to the difficult of maintaining the skill of microscopist, especially in the low transmissiong areas. RDT uses antibodies to detect one or several parasite-specific antigens in a drop of fresh blood. They do not require any special equipment. Therefore, RDT is suitable for primary health care institutions with limited facilities and unskilled staff. However, they may also fail to accurately diagnose for cases with low parasitaemia, and false positives are possible due to cross reactions [6–9].

Globally, it has been estimated that 276 million RDTs for malaria were sold in 2017, and the number rose to 412 million in 2018 [1]. RDT is being used more and more, regardless of the transmission setting. In sub-Saharan Africa, RDT has now become the most widely-used method for malaria diagnosis among suspected patients in public healthcare institutions [2]. However, previous economic evaluations of RDT were mainly performed in Africa, there was very little evidence from the elimination setting [10]. Moreover, many factors that impact the result of a cost-effectiveness analysis (CEA), such as incidence rate, the distribution of *Plasmodium* species, labor cost, and health workers’ awareness of malaria, were very different in different areas [11, 12]. And previous cost-effectiveness researches barely paied the attention to evaluating the cost of FP case [13–16].

Recent year, more and more countries set elimination as the goal of national malaria program, and many of them with zero indigenous case reported, such as Malaysia, China, Iran, El Salvador [1], it is urgent to know whether RDT is still cost-effective compared to microscopy in malaria elimination setting, and how the cost of FP cases affects the result of cost-effectiveness analysis. For filling these evidence gap, this study conducted a cost-effectiveness of the malaria diagnostic strategies from the joint perspective of health sector and patient, with the real-world data from China in 2018 based on a decision analytical model.

## Methods

### Study site

Jiangsu Province is a coastal area in East China. The malaria incidence rate there was about 250 cases per 1000 population at risk in the 1960s [17]. After decades-long efforts, there has been no indigenous case in Jiangsu since 2011. However, imported *Plasmodium* infections in this area have been increasing with the development of international trade, which poses tremendous thread for elimination [18–20]. In 2018, there were 243 imported malaria cases reported, which increased by 1.67% compared to 2017 (239 cases). All of them were adults, and the majority were male migrant workers who had been returned from sub-Saharan Africa with *P. falciparum* infections [19].

### Diagnostic strategies

Three strategies of malaria diagnostic testing (MDT) were compared in the model. Three types of febrile patients whose body temperature exceeded 38.5 degrees celsius would be involved in the malaria diagnostic testing, including 1) malaria case diagnosed according to clinical symptoms, 2) suspected malaria cases, 3) patients with unexplained fever. In the first strategy (MDT1), these febrile patients would undergo microscopy test, and patients with a positive result would be diagnosed as malaria. In the second strategy (MDT2), RDT were used, and diagnosis would be made based on the test results. In the last strategy (MDT3), patients would be tested using RDT at first, and those with a positive result would be followed by microscopy examination. If the results of microscopy were still positive, they would be confirmed as malaria.

### Decision-analytic model

**Fig. 1:**
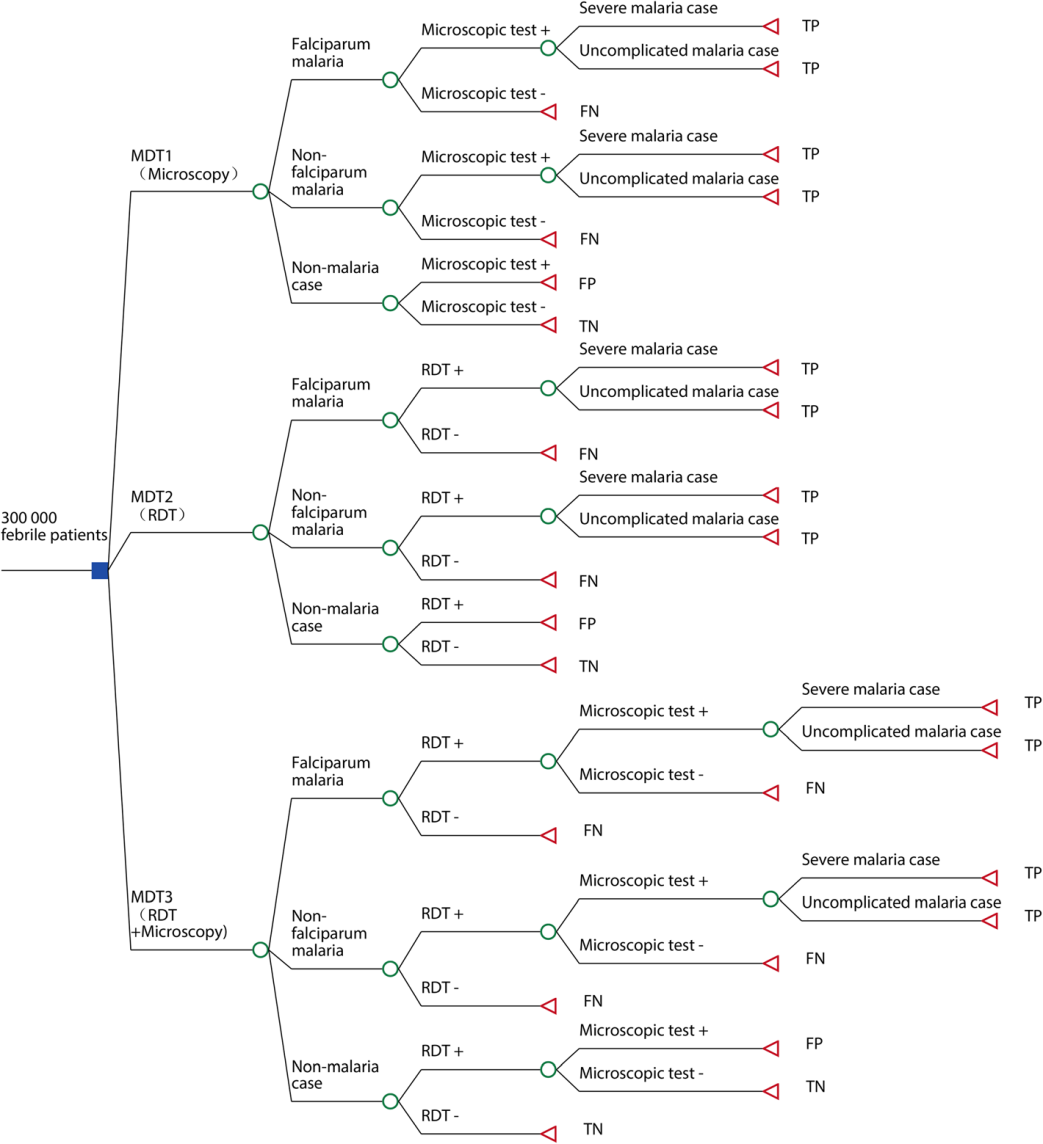
Basic structure of decision tree. +: Positive; -: Negative; FN: False-negative; FP: False-positive; TN: True-negatives; TP: True-positives

To compare the three malaria diagnosis strategies, a decision tree model was developed using TreeAge Pro software (Version 2019 - R1.1, TreeAge Software, LLC, Williamstown, United States). Figure 1 presents the basic structure of the decision tree. A hypothetical cohort of 300 000 febrile patients were simulated which is approximately the annual number of febrile patients who need blood tests in Jiangsu. Patients could either have malaria or not. The number of malaria cases was determined by the prevalence of malaria among febrile patients. Patients with malaria and positive diagnosis test results were considered as true positives. Treatment for different malaria status (uncomplicated and severe malaria cases) was assumed to be implemented according to the national malaria treatment guidelines. Specifically, uncomplicated malaria patients caused by *P. falciparum* would receive artemisinin-based combination therapies (ACTs) such as dihydroartemisinic and piperaquine; uncomplicated malaria patients caused by non-*P. falciparum* would receive chloroquine alone, or with primaquine (for vivax and ovale malaria); and all severe malaria patients would receive artemisinin injection such as artesunate. A part of uncomplicated malaria patients would be treated as intpatient, but all severe malaria patients would be treated as inpatient.

### Measurement of effect

Considering that the early detection of malaria cases in areas approaching elimination had a high priority, the effect of the MDT strategies was measured by the number of appropriately diagnosed malaria cases (true-positives, TP) in this study. The terminal nodes marked by TP in the decision tree (Fig. 1) were considered as the effect defined by this study.

### Measurement of cost

Costs in a year (2018) were measured from the health institution and patient joint perspective. Since all costs occurred within 1 year, they were not discounted. Costs were presented in Chinese Yuan (CNY) but then converted to US dollar (USD). And this study used 2018 yearly average currency exchange rate: CNY 6.6174 = USD 1.

Direct costs were categorized as direct medical costs and direct non-medical costs. The direct medical costs included the costs of malaria diagnosis testing (RDT or microscopy), the costs of antimalarial drugs, and other medical costs. The direct non-medical costs were the travel costs for the patients.

The costs of malaria diagnosis included material costs and labor costs of laboratory personnel while the costs of antimalarial drugs differed according to the type of plasmodium and the severity of patient’s symptoms.

The costs for false-positive (FP) and false-negative (FN) patients were taken into account according to clinical treatment. FP and FN patients have the same costs (i.e., the costs of malaria diagnosis tests, and antimalarial drugs) compared to those true-positives (TP) or true-negatives (TN) as they shared the same clinical pathways in the decision tree. But a FN patient in one strategy would incur an additional cost, the value of which is equivalent to all medical cost of one severe malaria case in the same strategy, including the cost of diagnosis, antimalarial drugs, and other treatments.

Other medical costs included registration costs, supplementary drug costs (eg, anti-fever medicines, Chinese patent medicines), biochemical diagnosis costs (except the malaria diagnosis test), bedside care costs (only for inpatient).

### Data source

Costs data from multiple sources were used, such as key informant interviews, hospital information systems (HIS), and patient surveys. The costs for the antimalarial drugs and RDT were made based on key informant interviews with healthcare administrators. According to the national health policy in China, both antimalarial drugs and RDT were purchased and distributed to healthcare facilities by health administrative department, and they were all free for patients. So, the key informant interviews with experts were conducted to estimate their costs. And microscopy test had been kept at extremely low price for patients due to government subsidies. Its costs was also estimated using key informant interviews.

Inpatient costs were estimated based on the data of 25 latest malaria cases in 2018 identified through the HIS of a designated hospital for malaria treatment in Jiangsu province. Outpatient costs were collected from confirmed malaria cases reported between the first week and the forty-ninth week of 2018 via telephone surveys conduceted by one researcher. Each phone number was contacted no more than three times. Cost information was used only if the patient answered the call and gave consent to participate. Transportation costs were also collected by telephone surveys.

Epidemiological data were obtained from malaria surveillance reports, such as the proportion of falciparum malaria, and the proportion of hospitalization for uncomplicated malaria cases. The accuracy of MDT was derived from published literature [21–25].

### Cost-effectiveness analysis and sensitivity analysis

In deterministic cost-effectiveness analysis, total costs of the cohort were calculated separately for each strategy. Incremental cost-effectiveness ratio (ICER) compared the incremental costs that one strategy would incure over another for one additional malaria case that have been appropriately diagnosed and treated.

To examine the uncertainty brought by the underlying assumptions, a series of one-way sensitivity analyses, including all parameters, were conducted [26]. Moreover, a two-way sensitivity analysis based on the table of value sets in TreeAge software was undertaken to reveal the impact of different proportion of falciparum malaria among all malaria cases. This method was different with normal two-way sensitivity analysis (two variables change in the same direction). The parameter sets in this research would keep the total incidence of malaria fixed while the proportion of falciparum malaria changed from 50 to 100%. That meaned when the incidence of falciparum malaria increased, the incidence of non-falciparum malaria decreased by the same value. This range include all fluctuations in the proportion of imported malaria species in China in the past decade [3].

In order to reflect the real situation, total costs and effects for each strategy were estimated by Monte Carlo simulation, a probabilistic sensitivity analysis (PSA) method. Monte Carlo simulation was conducted to incorporate uncertainties of multiple parameters into an analysis by assigning statistical distributions to all relevant parameters. The distributions were assigned to parameters considering the data uncertainty caused by statistical methods and the forecast of cost fluctuations [27]. Beta distribution was assigned to the sensitivity of RDT to make sure it could be constrained between zero and one [26, 28]. Triangular distribution was used for the sensitivity of microscopy, as it was not likely to follow a normal or beta distribution. Gamma distribution was specified for selected cost parameters to capture their strictly-positive and right-skewed nature [16, 28]. Uniform distribution was also used to costs parameters according to the intervals estimated by the key informants [16]. Monte Carlo simulation would generate random draws from these distributions and run 1000 iterations. Uncertainty would be presented in a figure of incremental cost-effectiveness plane.

## Results

In terms of diagnosis cost, this research found that the average cost of diagnosis by RDT was USD 2.19 per test, including material cost (USD 1.51) and labor cost (USD 0.68). The average cost of diagnosis by microscopy was USD 6.98 per test, including material cost (USD 0.18) and labor cost (USD 6.80, Table 1). In terms of treatment cost, the average cost of oral medication for malaria outpatient was USD 6.04 (chloroquine and primaquine) or 4.53 (dihydroartemisinic and piperaquine). The average cost of artesunate injection for malaria inpatient was USD 132.98. In addition to the cost of malaria diagnosis and antimalaria treatment, other medical costs incurred by outpatient was USD 31.58, mainly including registration and other diagnostic test. Other medical costs incurred by inpatient with uncomplicated malaria was USD 1167.83, mainly including supplementary medication and bedside care. Other medical costs incurred by inpatient with severe malaria was USD 17 569.29, mainly including diagnosis and treatment of complications, bedside care. The incidence of falciparum malaria and non-falciparum malaria per 1000 febrile patients was respectively 0.71 and 0.17 (Table 2). More details about cost parameters and epidemiological parameters were shown in Table 1 and 2.

**Table 1:** Cost components and unit costs

Items	Base case value (USD)	Range for one-way sensitivity analysis
**Direct medical cost**		
**1) Malaria diagnosis**		
RDT - Malaria Pf/Pan Whole Blood Test per test	1.51 ^a^	1.21–2.27
Microscopy - Material cost of thick smear per exam	0.18 ^a^	0.15–0.30
Labor cost of laboratory staff per hour	6.80 ^a^	3.02–11.33
Time spent on RDT per test	0.1 (hour) ^a^	0.08–0.25
Time spent on thick smear test per test	1 (hour) ^a^	0.5–1.5
**2) Malaria treatment**		
Chloroquine and primaquine per course of treatment	6.04 ^a^	6.04–7.56
Dihydroartemisinic and piperaquine per course of treatment	4.53 ^a^	4.53–6.80
Artesunate injection per course of treatment	132.98 ^a^	132.95–151.12
**3) Other relative diagnosis and treatment**		
Other medical costs for outpatient per uncomplicated malaria case	31.58 ^b^	30.22–45.34
Other medical costs for inpatient per uncomplicated malaria case	1167.83 ^c^	824.49–1511.17
Other medical costs per severe malaria case	17 569.29 ^c^	10 578.17–45 335.03
Other medical costs per false positive case	786.34 ^c^	435.35–1137.33
Other medical costs per false negative case	11 652.67 ^c^	7555.84–22 667.51
**Direct non-medical cost**		
Travel cost of patient visiting health care sector per person	3.02 ^b^	1.51–4.53

^a^ Data collected by key informant interview, ^b^Data collected by patient survey, ^c^Data collected from hospital information system

*RDT* Rapid diagnostic test, *USD* United States Dollar

**Table 2:** Epidemiological parameters considered in the analytic model

Parameter	Base case value	Range for one-way sensitivity analysis
*falciparum* cases per 1000 febrile patients	0.7073 ^a^	0.3537–1.0610
Non*-falciparum* cases per 1000 febrile patients	0.1746 ^a^	0.0873–0.2619
Probability of conversion of *falciparum* malaria into severe case	3% ^b^	1–5%
Probability of conversion of non-*falciparum* malaria into severe case	0.10% ^b^	0.05–0.5%
Proportion of inpatient in all uncomplicated malaria cases	66% ^b^	20–80%
Sensitivity of RDT for *falciparum malaria*	93% [21–25]	91–93%
Sensitivity of RDT for non-*falciparum malaria*	91% [21–25]	89–92%
Specificity of RDT	99% [21–25]	98–99%
Sensitivity of microscopy	90% ^c^	85–95%
Specificity of microscopy	100% ^c^	90–100%

^a^Data collected from malaria surveillance reports, ^b^Data collected by patient survey, ^c^Data collected by key informant interview

*RDT* Rapid diagnostic test

The results of deterministic cost-effectiveness analysis based on the base case value (Tables 1, 2) are shown in Table 3. MDT2 (RDT) had the highest number of appropriately diagnosed and treated malaria cases (245 cases) compared with MDT1 (238 cases) and MDT3 (221 cases) but it also had the highest costs (about 4.47 million USD). No strategy was dominated.

**Table 3:** The results of cost-effectiveness analysis

Strategy	MDT3	MDT1	MDT2
	(RDT and Microscopy)	(Microscopy)	(RDT)
**Cost (USD)**	2 754 254.53	3 626 228.08	4 465 725.40
**Effect (case)**	220.5	238.11	245
**Cost/Effect**	12 491.08	15 229.33	18 227.64
**Incremental cost**	-	871 973.55	1 711 471.87
**Incremental effect**	-	17.61	24.5
**ICER**	-	49 514.29	69 856.70

Note:*MDT* Malaria diagnostic testing, *RDT* Rapid diagnostic tests, *USD* United States Dollar, *ICER* Incremental cost-effectiveness ratio

**Fig. 2:**
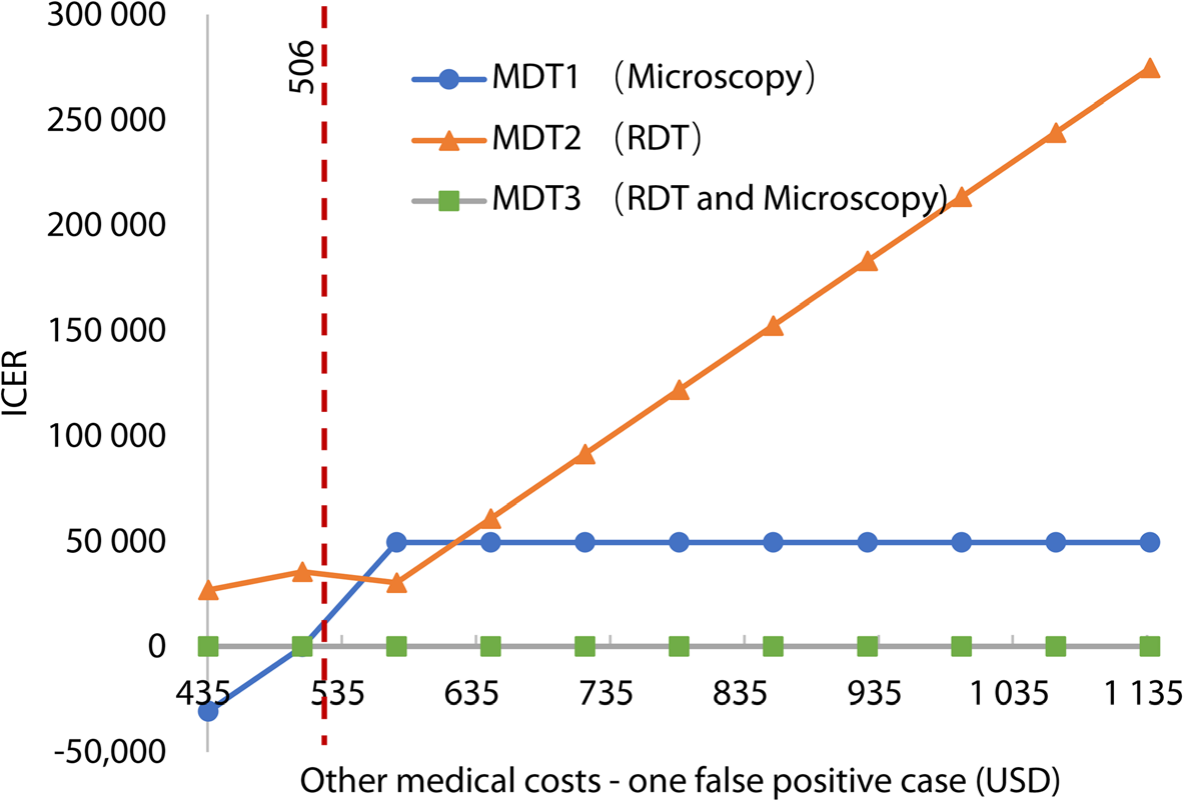
Sensitivity analysis of other medical costs – one false-positive case (USD)

For one-way sensitivity analysis of cost parameters, the CEA result was robust to most of parameters in the range (Table 1), except the labor cost of laboratory staff, and other medical costs for false-positive cases. Table 4 showed that ICER was sensitive to varying labor cost of laboratory staff. When the labor cost (per hour) was USD 3.52, MDT3 was dominated. However, when the labor cost increased to USD 10.17 per hour, MDT1 was dominated. When labor cost fluctuated around USD 6.80, such as USD 5.18, 6.84, or 8.50, the results of sensitivity analysis were similar to the base-case results. No strategy was dominated. Figure 2 showed that when other medical costs for one FP case were lower than USD 506, MDT1 was dominated. ICER in MDT2 was found to be more sensitive to the changes in the other medical costs of one FP case.

**Table 4:** One-way sensitivity analysis of labor cost of laboratory staff

Labor Cost (USD, per hour)	Strategy	Cost (USD, million)	Effect (case)	C/E	Increment Cost	Increment Effect	ICER
3.52	MDT1	2.642	238	0.011	-	-	-
	MDT3^a^	2.645	220	0.012	0.003	-18	-0.0002^a^
	MDT2	4.367	245	0.018	1.726	7	0.2505
5.18	MDT3	2.7	220	0.012	-	-	-
	MDT1	3.14	238	0.013	0.44	18	0.025
	MDT2	4.417	245	0.018	1.277	7	0.1853
6.84	MDT3	2.756	220	0.012	-	-	-
	MDT1	3.639	238	0.015	0.883	18	0.0502
	MDT2	4.467	245	0.018	0.828	7	0.1202
8.5	MDT3	2.811	220	0.013	-	-	-
	MDT1	4.138	238	0.017	1.327	18	0.0753
	MDT2	4.517	245	0.018	0.379	7	0.0551
10.17	MDT3	2.866	220	0.013	-	-	-
	MDT2	4.567	245	0.019	1.701	24	0.0694
	MDT1^a^	4.636	238	0.019	0.069	-7	-0.0101^a^

Note: ^a^ The strategy that was dominated by others

*USD* United States Dollar, *C/E* Cost/effect, *ICER* Incremental cost-effectiveness ratio, *MDT* Malaria diagnostic testing

For one-way sensitivity analysis of cost epidemiological parameters in Table 2, the deterministic CEA results was robust to the sensitivity and specificity of RDT, but can be influenced by the sensitivity and specificity of microscopy. If the sensitivity of microscopy was above 92.58%, the MDT1 would have the highest effect, and MDT2 would be dominated by MDT1. If the specificity of microscopy was below 99.1%, the MDT1 would be dominated by MDT2.

**Fig. 3:**
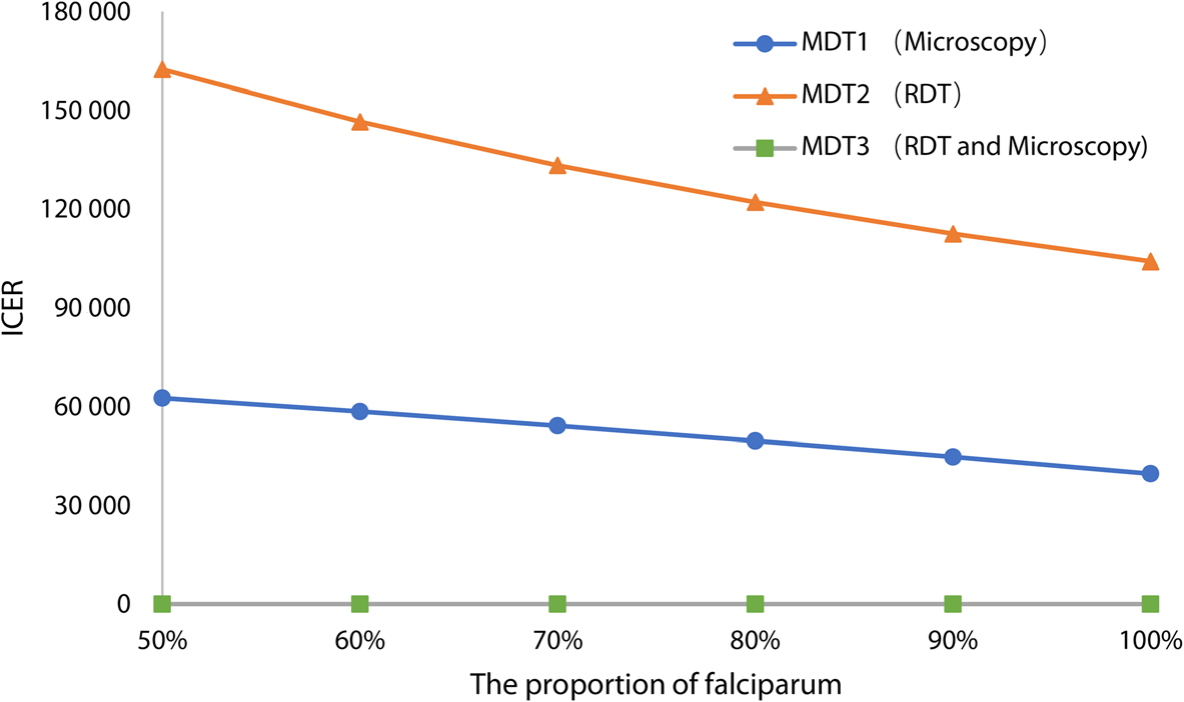
Sensitivity analysis of the proportion of falciparum. MDT: Malaria diagnostic testing; RDT: Rapid diagnostic test

For two-way sensitivity of the proportion of falciparum malaria and non-falciparum malaria, while the annual incidence of malaria was low and stable (Table 5), the CEA result was not sensitive to the change in the proportion of *falciparum*. Although ICERs decreased when the proportion of *falciparum* increased (Fig. 3).

**Table 5:** Value set of incidence in sensitivity analysis for the proportion of *falciparum* malaria

**The proportion of** ***falciparum malaria*** **simulated in model**	**50%**	**60%**	**70%**	**80%**	**90%**	**100%**
The incidence of *falciparum* malaria	0.441	0.529	0.617	0.706	0.794	0.882
(per 1000 febrile patients)						
The incidence of Non-*falciparum* malaria	0.441	0.353	0.265	0.176	0.088	0
(per 1000 febrile patients)

**Fig. 4:**
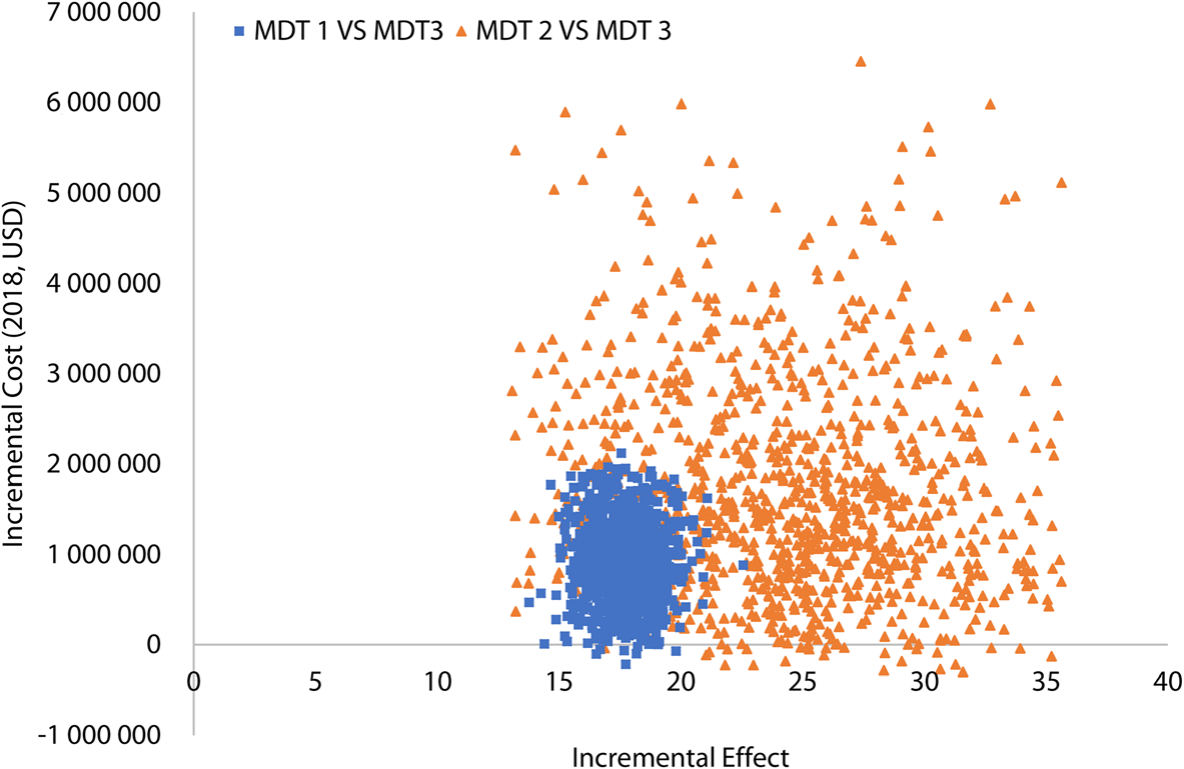
Incremental cost-effectiveness plan for Monte Carlo simulations for MDT1 versus MDT3 and MDT2 versus MDT3. MDT: Malaria diagnostic testing

**Fig. 5:**
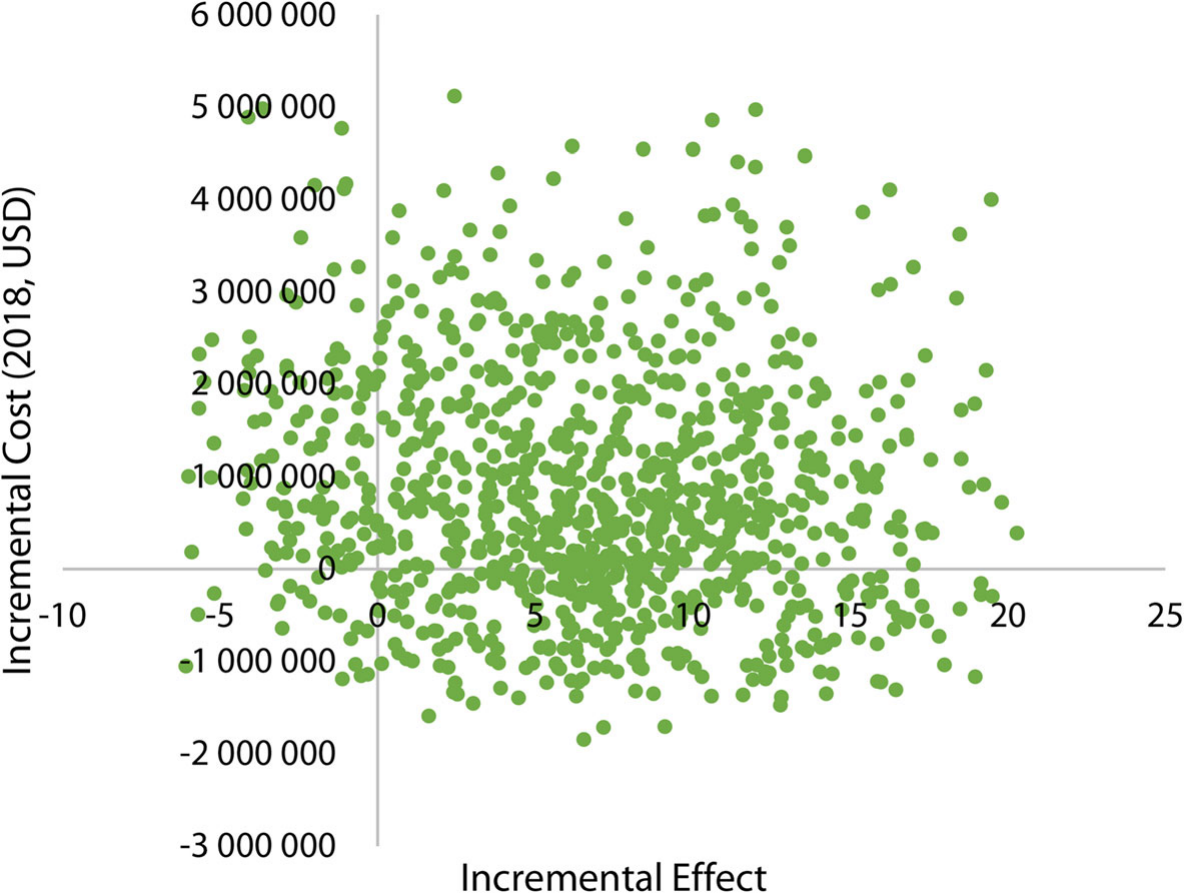
Incremental cost-effectiveness plan for Monte Carlo simulations for MDT2 versus MDT1. MDT: Malaria diagnostic testingpd

For probabilistic sensitivity analysis, based on the distributions of key parameters (Table 6), the incremental cost-effectiveness (ICE) plane was made to show the result of 1000 Monte Carlo simulations for MDT1 versus MDT3 and MDT2 versus MDT3 (Fig. 4). The majority of simulations for MDT1 compared with MDT3 were in the northeast quadrant, indicating that MDT1 resulted in higher effect at an increasing cost. And the simulations for MDT2 compared with MDT3 were in the same quadrant, indicationg that MDT2 also resulted in higher effect at an increasing cost. But the distributions of two series of scatter points overlapped in Fig. 4. So Fig. 5 was made to show the ICE plan for simulations for MDT2 versus MDT1. In Fig. 5, there were 61.2% scatter points in northeast quadrant, 26.3% in southeast, 20.0% in southwest, and 10.5% in northwest, indicating that MDT2 resulted in higher effect at an increasing cost whit a high probability (61.2%).

**Table 6:** Parameters and distributions for Monte Carlo simulation

Parameters	Distribution	PSA parameters in Treeage		
RDT - Malaria Pf/Pan Whole Blood Test per test	Uniform	Low = 1.21	High = 1.51	
Travel cost of patient visiting health care sector per person	Triangular	Min = 0.30	Likeliest = 3.02	Max = 6.35
Labor cost of laboratory staff per hour	Uniform	Low = 6.80	High = 7.56	
Other medical costs for outpatient per uncomplicated malaria case	Gamma	Mean = 31.58	SD = 88.4	
Other medical costs for inpatient per uncomplicated malaria case	Gamma	Mean = 1167.83	SD = 604.00	
Sensitivity of RDT for *Plasmodium falciparum*	Beta	Mean = 0.93	SD = 0.02	
Sensitivity of RDT for non-*Plasmodium* falciparum	Beta	Mean = 0.91	SD = 0.03	
Specificity of RDT	Beta	Mean = 0.99	SD = 0.005	
Sensitivity of microscopy	Triangular	Min = 0.85	Likeliest = 0.90	Max = 0.95
Specificity of microscopy	Triangular	Min = 0.99	Likeliest = 1.00	Max = 1.00
Time spent on RDT per test	Triangular	Min = 0.08	Likeliest = 0.1	Max = 0.25
Time spent on thick smear test per test	Triangular	Min = 0.50	Likeliest = 1.00	Max = 1.50

*PSA* Probabilistic sensitivity analysis, *RDT* Rapid diagnostic tests, *SD* Standard deviation

## Discussion

The CEA results suggested that MDT2 (RDT) has more effect than MDT1 (microscopy) and MDT3 (RDT followed by microscopy) with the higher cost in elimination setting. The part of result, that RDT had more effect than microscopy, was in line with two similar researches in Afghanistan and Uganda [15, 29]. But They indicated that RDT had lower cost than microscopy. There was still a difference between this research and theirs. It was that they did not calculated the cost of treating FP and FN cases into the total cost. But in this research, the total costs for each strategy consisted of the diagnosis cost and the treatment cost of FP and FN cases. In area without local transmission or very low transmission, the small gap in specificity between microscopy and RDT would be amplified by the large number of non-patients. Our results indicated that the other medical costs (excluding the malaria diagnosis and treatment) of FP cases was the main cost for the higher total cost of MDT2 when it was compared to either MDT1 or MDT3. Although the diagnosis cost per febrile ptient in MDT2 was lower than the other two strategies. There were more FP cases in MDT2 because of its slightly lower specificity, which causes the more unnecessary treatment costs and higher total cost. Now in Jiangsu, many hospitals tended to treat malaria case as inpatient. From the doctor’s perspective, hospitalization meant better compliance, and lower retransmission risks. From the patient’s perspective, hospitalization meant that part of medical expenses could be reimbursed by insurance. So this tendency was the common intention of both doctors and patients, and it actually made MDT2 more costly than other strategies due to more FP treated as inpatient.

Since the results also indicated that the total costs of MDT2 was sensitive to the other medical costs of FP cases, it was suggested that MDT2 could be a more cost-effective strategy if the number and costs of FP cases could be controlled. There were many measures that could be taken, such as re-testing by other more accurate diagnositic technologies, restricting its use in febrile patients without travel history in endemic areas, and reducing the hospitalization of mild patients. Therefore, this study suggested setting standards for hospitalization of malaria case would control the total cost of RDT strategy.

If all the patients with RDT positive results could be retested by microscopy immediately, like MDT3, most FP cases could be avoided. However, meanwhile, some patients with malaria would be missed diagnosis due to low sensitivity of microscopy. Compared with local center for disease control and prevention (CDC), the maintenance of microscopy capability to detect malaria in medical institutions is more difficult and the cost of training was higher. So we suggested that all blood samples of patients with positive RDT in hospitals should be redetected by microscopy or mocecular detection techniques, such as polymerase chain reaction (PCR) or loopmediated isothermal amplification (LAMP), in CDC. The county-level CDC In China was responsible for microscopic review of malaria cases reported by medical institutions. PCR or LAMP were used by CDC to improve the sensitivity and identify the species of *Plasmodium*. This was also a part of content of the 1-3-7 malaria surveillance and response strategy in China [30]. If we could detect FP cases timely in this way, and terminate wrong treatment, RDT would be more cost-effective, and even dominate microscopy, when other medical costs of FP case are under USD 506 based on our results (Fig. 2).

The sensitivity of microscopy depended on the skill level and proficiency of laboratory personnel. In areas without local transmission, doctors may encounter few imported malaria cases. It was challenging to maintain the malaria diagnosis skills in China’s primary health care, such as Township Health Centers (THCs) and Community Health Centers (CHCs). This was a common situation for most countries in malaria elimination or malaria-free phase. Continuous training was necessary to maintain the skill level of laboratory personnel. Even so, in most THCs, CHCs or hospitals in elimination setting, the high sensitivity of the microscopy was still difficult to maintain [31–33]. Therefore, the sensitivity and specificity of the microscopy were less likely to reach the thresholds which can make microscopy dominant to RDT. Moreover, with the economic development, laboratory personnel salary in general hospitals in Central city was close to the upper limit of the range adopted in the sensitivity analysis. The results of one-way sensitivity analysis also indicated that RDT would dominate microscopy in other specific scenarios, such as, when the proficiency of laboratory personnel decreased, and salary increased. Than the image recognition based on artificial intelligence is likely to reduce labor costs and maintain the accuracy of microscopy [34, 35]. But for now, it was difficult to for microscopy to have a higher sensitivity compared to RDT. Considering the potential local transmission risk caused by FN cases, we suggested that RDT should be the first choice in the area targetting for eliminaiton.

There were huge differences in the proportion of *Plasmodium* species among CEA researches [12, 15, 16]. In elimination setting, the epidemiological characteristics of imported malaria case was not impacted by the local climate or the area where anopheles were active, but impacted by the areas where imported malaria cases came from. In China, the main factor that has impact on imported cases was the return of migrant workers. The proportion of *falciparum* in all malaria cases was constantly changing each year. Therefore, sensitivity analysis was performed to evaluate the robustness of CEA results to this kind of changes in the proportion of *falciparum*. The result indicated that MDT2 (RDT) always had more effect than other strategies, when the proportion of *falciparum* varied between 50 and 100%.

This research offered evidence with a realistic vision for the area in the elimination setting, where the resources still need to be continuously invested in order to achieve and maintain malaria-free. The evidence would inform decision makers that an effective sustainable surveillance system could help to avoid the cost of treating FP cases, and then make RDT more cost-effective.

This study had the limitation in the collection of medical cost data. The best plan was to connect to all of the hospitals that treated malaria cases in 2018, and extract medical cost information from their HIS. However, due to resource limit and time constraints, we could’t receive consent from all of the hospitals. For ameliorating the potential impact of this limitation on the results, we assigned the credible range, that was calculated by standard statistical methods, to each cost parameter in PSA.

## Conclusions

The cost-effectiveness analysis suggested that MDT2 (RDT) strategy has the higher effects and higher total cost compared with MDT1 (microscopy) and MDT3 (RDT followed by microscopy) in the setting of malaria elimination. These results were robust to the majority of cost parameters in sensitivity analysis, except labor costs and treatment costs for FP case. RDT would be the dominant strategy if the treatment costs of FP cases could be controlled or when labor costs were higher than USD 10.17 per hour.

## Data Availability

The datasets used and analyzed during the current study are available from the corresponding author on reasonable request.

## Abbreviations

ACTs: Artemisinin-based combination therapies
CDC: Center for disease control and prevention
CEA: Cost-effectiveness analysis
CER: Cost-effectiveness ratio
CHCs: Community Health Centers
CNY: Chinese Yuan
FN: False-negative
FP: False-positive
HIS: Hospital information system
ICER: Incremental cost-effectiveness ratio
ICE: Incremental cost-effectiveness
LAMP: Loopmediated isothermal amplification
MDT: Malaria diagnostic testing
PCR: Polymerase chain reaction
PSA: Probabilistic Sensitivity Analysis
RDT: Rapid diagnostic test
THCs: Township Health Centers
TN: True-negatives
TP: True-positives
USD: United States Dollar
WHO: World Health Organization
